# Phase I and pharmacokinetic study of the vascular‐disrupting agent CKD‐516 (NOV120401) in patients with refractory solid tumors

**DOI:** 10.1002/prp2.568

**Published:** 2020-03-12

**Authors:** Hark Kyun Kim, Jeong Won Kang, Young‐Whan Park, Jung Young Kim, Minchae Kim, Soo Jin Kim, Se‐mi Kim, Keun Ho Ryu, Seonghae Yoon, Yun Kim, Joo‐Youn Cho, Keun Seok Lee, Tak Yun, Kiwon Kim, Mi Hyang Kwak, Tae‐Sung Kim, Jinsoo Chung, Joong‐Won Park

**Affiliations:** ^1^ National Cancer Center Hospital Goyang‐si Gyeonggi‐do Republic of Korea; ^2^ National Oncoventure National Cancer Center Goyang‐si Gyeonggi‐do Republic of Korea; ^3^ CKD Research Center Yongin‐si Gyeonggi‐do Republic of Korea; ^4^ Clinical Trials Center Seoul National University Bundang Hospital Bundang‐gu Seongnam‐si Republic of Korea; ^5^ Department of Clinical Pharmacology and Therapeutics Seoul National University College of Medicine Seoul Republic of Korea

**Keywords:** cancer, phase 1 trial, vascular

## Abstract

We report a phase I pharmacological study of an oral formulation of CKD‐516, a vascular‐disrupting agent, in patients with refractory solid tumors. Twenty‐seven patients (16 in the dose‐escalation cohort and 11 in the expansion cohort) received a single daily dose (5‐25 mg) of CKD‐516 five days per week. Nausea (67%) and diarrhea (63%) were the most common treatment‐related adverse events. The recommended phase II dose of oral CKD‐516 was 20 mg/d (15 mg/d with a body surface area (BSA) <1.65 m^2^). Notably, S‐516 half‐lives in patients receiving 15‐20 mg CKD‐516/d significantly differed between patients with and without splenomegaly that is suggestive of portal hypertension associated with liver cirrhosis (6.1 vs 4.6 hours, respectively). Of 11 patients without splenomegaly who completed at least one cycle of a daily CKD‐516 dose of either 15 or 20 mg, only one patient (9.1%) suffered from any dose‐limiting toxicity. We conclude that a daily oral dose of 15 or 20 mg CKD‐516 five days per week could be tolerable in patients without liver cirrhosis.

**Abbreviations**
ALT: alanine transaminase
CBC: complete blood count
CT: computed tomography
DLT: dose‐limiting toxicities
ECG: electrocardiography
ECOG: Eastern Cooperative Oncology Group
PET: Positron emission tomography
ULN: upper limit of normal
VDAs: Vascular‐disrupting agents

## 1 INTRODUCTION

Vascular‐disrupting agents (VDAs) are a relatively new class of drugs that act on the established tumor vasculature, causing hypoxia and necrosis of the tumor mass.^1^, ^2^, ^3^, ^4^, ^5^, ^6^ Tubulin colchicine binding site inhibitor VDAs have been most extensively tested in clinical trials.^3^, ^4^, ^5^, ^6^ Common toxicities of these agents include nausea, diarrhea, tumor pain, and hypertension.^3^, ^4^, ^5^, ^6^ CKD‐516 (NOV120401), developed by Chong Kun Dang (Seoul, Korea), is a VDA prodrug that is rapidly converted into the active moiety, S‐516.^7^, ^8^, ^9^, ^10^ S‐516 possesses strong antitumor activity and can increase the antitumor activities of various chemotherapeutic and/or immunotherapeutic regimens.^7^, ^8^, ^9^, ^10^ CKD‐516 was shown to exhibit a manageable safety profile in patients with solid tumors in an intravenous injection clinical study (NCT01028859).^11^ Using doses up to 9 mg/m^2^ (days 1 and 8, every 3 weeks), intravenous CKD‐516 resulted in no dose‐limiting toxicities (DLT); however, a weekly intravenous infusion of 12 mg/m^2^ CKD‐516 led to two cases of grade 3 hypertension, considered a DLT.^11^ A subsequent study (NCT01560325) demonstrated that more frequent dosing of CKD‐516 was not associated with increases in toxicity. An oral formulation of CKD‐516 was developed to improve its efficacy and patient compliance. Here, we report a phase I pharmacological study to determine the optimal dosing regimen for oral CKD‐516 in patients with refractory solid tumors.

## 2 MATERIALS AND METHODS

This was a single‐institution, phase I study conducted at the National Cancer Center Korea (ClinicalTrials.gov identifier, NCT02300467). All patients provided informed consent for the study protocol, which was approved by the National Cancer Center Institutional Review Board (NCCIRB 2014‐0174). Eligibility criteria included age greater than or equal to 19 years; Eastern Cooperative Oncology Group (ECOG) performance score less than or equal to 2; and adequate bone marrow, renal, and hepatic function [absolute neutrophil count ≥ 1500/mm^3^, platelet count ≥ 100 000/mm^3^, hemoglobin ≥ 9.0 g/dL, serum creatinine level ≤ 1.5 g/dL, or a calculated creatinine clearance ≥ 50 mL/min, aspartate transaminase (AST) and alanine transaminase (ALT) ≤3 × the upper limit of normal (ULN) or ≤ 5× the ULN in cases of liver involvement, bilirubin ≤ 1.5 × the ULN, and prothrombin time and activated partial thromboplastin time ≤ 1.5 × the ULN]. Patients were required to have histologically or cytologically confirmed cancers that were refractory to at least 1 therapeutic regimen for metastatic disease. In cases of hepatocellular carcinoma (HCC), histological confirmation was not required if α‐fetoprotein (AFP) levels were greater than or equal to 200 ng/mL, radiographic findings were consistent with HCC, and either HBsAg or anti‐HCV was present. Previous chemotherapy was discontinued at least 2 weeks before study entry.

### 2.1 Dosing schedule

After fasting or at least 2 hours after meal, oral CKD‐516 tablets were administered to patients on days 1‐5, 8‐12, and 15‐19 of a 3‐week cycle (ie, 5 days on and 2 days off for better patient adherence). Treatment was planned for 12 cycles unless disease progression or unacceptable toxicity developed. The first dose for humans was 5 mg/d, which is one‐tenth the human equivalent dose of the maximum tolerated dose in female rats (5 mg/kg/d), as calculated during a 2‐week repeated‐dose toxicity test. A traditional 3 + 3 dose‐escalation design was used for the dose‐escalation phase. The expansion cohort was initially treated with a dose of 20 mg/d. Due to the DLT observed in the first patient, the study protocol was modified after 1 patient was treated so that the daily doses were 15 mg/d and 20 mg/d for a BSA less than and greater than or equal to 1.65 m^2^, respectively.

### 2.2 Safety and efficacy assessments

Pretreatment evaluations consisted of a physical examination, an assessment of ECOG performance status and vital signs, 12‐lead electrocardiography (ECG), a complete blood count (CBC), and blood chemistry levels. Positron emission tomography (PET) scans were performed to evaluate baseline disease status. Follow‐up safety evaluations were performed at the beginning of each treatment cycle and at study termination, and included a physical examination, an assessment of vital signs and ECOG performance status, a CBC, and blood chemistry levels. Adverse events and evidence of toxicity were evaluated according to National Cancer Institute Common Criteria for Adverse Events (version 4.03). A DLT was defined as grade 4 neutropenia lasting more than 7 days, grade 3 or 4 neutropenic fever (>38.3°C), grade 4 thrombocytopenia, grade 3 thrombocytopenia with bleeding, or any treatment‐related grade 3 or 4 nonhematologic toxicity. Grade 3 or 4 nausea, vomiting, diarrhea, and tumor pain lasting fewer than 3 days with symptomatic management were not considered DLTs. The following nonhematologic toxicities were considered DLTs if they persisted for longer than 7 days: grade 3 fatigue, hypertension (>140 mmHg/90 mmHg), and an elevation of AST or ALT > 5× the ULN.

Treatment response was assessed by computed tomography (CT) 2 weeks before the first dose and every 8 weeks thereafter. CT was used to evaluate the presence of splenomegaly (defined as an enlargement of the spleen > 12 cm along the longest diameter), which is suggestive of portal hypertension associated with liver cirrhosis. Responses to therapy were evaluated primarily according to the RECIST guideline (v1.1).^12^ PET scans were repeated 8 weeks after the first dose.

### 2.3 Pharmacokinetics assessment

Sampling for the assessments of pharmacokinetics parameters was performed in 27 patients on days 1 and 19 of the first cycle of CKD‐516 administration. Blood samples were collected in heparin tubes before and 0.25, 0.5, 1, 1.5, 2, 3, 4, 6, 8, 12, and 24 hours after CKD‐516 dosing. Trough samples were also collected on day 5 of cycle 1. Plasma was separated by centrifugation (1620 *g* at 4°C for 15 minutes) within 5 minutes of collection and stored at −80°C. Plasma concentrations of CKD‐516, S‐516, and M9 (an inactive metabolite of CKD‐516) were determined using a protein precipitation method on a validated reverse‐phase ultra‐fast liquid chromatography system (Shimadzu, Kyoto, Japan) coupled with a QTRAP 5500 mass spectrometer (AB SCIEX, Framingham, MA) and electrospray ionization (ESI) interface. Peak concentrations of CKD‐516, S‐516, and M9 were determined according to comparisons with the internal standard (S‐516‐d_3_). The maximum drug concentrations in plasma (*C*_max_) and the time at which C_max_ was reached (*T*_max_) were determined directly from the observed values. The area under the curve (AUC) from the time of dosing to the last measurable concentration (AUC_last_) was calculated from the time‐concentration curves by a noncompartmental analysis using a linear/log‐linear trapezoidal method. The terminal half‐life (T_1/2_) of S‐516 was calculated as follows: T_1/2_ = ln(2)/λz, where λz is the terminal elimination rate constant. Linear regression analyses (power model) were conducted with log‐transformed pharmacokinetic parameters (*C*_max_ and AUC_last_) with the following equations:

$$\ln\left(C_{\max}\right)=\beta_{0}+\beta_{1}\times \text{ln(Dose)}$$

$$\ln\left({\text{AUC}}_{\text{last}}\right)=\beta_{0}+\beta_{1}\times \text{ln(Dose)}$$

The estimates of the regression parameter (*β_1_*) and its 2‐sided 95% confidence intervals (CI) were derived from the equations. Adjusted R^2^ was calculated between log‐transformed CKD‐516 dose and the log‐transformed pharmacokinetic parameters.

## 3 RESULTS

### 3.1 Enrollment

Twenty‐seven patients (16 in the dose‐escalation cohort and 11 in the expansion cohort) received at least one dose (5‐25 mg/d) of the study drug (Table 1). The median age of the patients was 55.7 years. Treatment was discontinued due to disease progression and toxicity in 19 (70.4%) and 7 (25.9%) patients, respectively, whereas one patient withdrew consent before the completion of the study. The average duration of drug administration was 31.9 days, and the average treatment cycle duration was 2.4 cycles. The average daily dose was 16.1 mg CKD‐516/d.

**Table 1:** Patient characteristics

	Dose escalation (n = 16)	Expansion (n = 11)
Age, median (range)	55 (22‐73)	58 (43‐71)
Male (%)	8 (50%)	7 (64%)
Primary tumor		
Breast cancer	5 (31%)	0
Colorectal cancer	3 (19%)	0
Soft tissue sarcoma	2 (13%)	0
Hepatocellular carcinoma	1 (6%)	5 (45%)
Renal cell carcinoma	1 (6%)	3 (27%)
Non–small cell lung carcinoma	1 (6%)	1 (9%)
Small cell lung carcinoma	1 (6%)	1 (9%)
Gastric cancer	1 (6%)	0
Nasopharyngeal carcinoma	1 (6%)	0
Malignant melanoma	0	1 (9%)
Previous systemic treatment regimen		
1	2 (13%)	4 (36%)
2	2 (13%)	3 (27%)
3	3 (19%)	2 (18%)
4 or more	10 (63%)	2 (18%)

### 3.2 Overall adverse events

Table 2 summarizes the treatment‐related adverse events that were observed in at least 10% of patients. Nausea/vomiting and diarrhea were the most common treatment‐related adverse events.

**Table 2:** Treatment‐related adverse events with a total incidence > 10%

	Grade 1	Grade 2	Grade 3	Grade 4	Total	
	n (%)	n (%)	n (%)	n (%)	n (%)	
Vomiting	9 (33%)	4 (15%)	4 (15%)	0	17	(63%)
Nausea	11 (41%)	3 (11%)	4 (15%)	0	18	(67%)
Diarrhea	13 (48%)	2 (7%)	2 (7%)	0	17	(63%)
Constipation	6 (22%)	0 (0%)	0	0	6	(22%)
Abdominal pain	5 (19%)	2 (7%)	1 (4%)	0	8	(30%)
Epigastric soreness	3 (11%)	1 (4%)	0	0	4	(15%)
Anorexia	9 (33%)	6 (22%)	0	0	15	(56%)
Dizziness	10 (37%)	0	0	0	10	(37%)
Fatigue	3 (11%)	6 (22%)	0	0	9	(33%)
Fever	8 (30%)	0	0	0	8	(30%)
Anemia	1 (4%)	7 (26%)	1 (4%)	0	9	(33%)
Neutropenia	0	0	4 (15%)	1 (4%)	5	(19%)
Thrombocytopenia	2 (7%)	1 (4%)	3 (11%)	1 (4%)	7	(26%)
Prolonged QT interval	7 (26%)	1 (4%)	1 (4%)	0	9	(33%)

### 3.3 Safety profiles in the dose‐escalation cohort

Sixteen patients were enrolled in the dose‐escalation cohort. At dose level 2 (10 mg/d), 1 patient was removed from the trial during the DLT evaluation period due to early disease progression. Hence, DLT was only evaluated in 15 patients (Table 3). At dose level 4 (25 mg/d), all 3 patients experienced DLTs [grade 3 nausea/vomiting, grade 3 paresthesia of the lower extremities (possibly due to a transient ischemic attack of the spinal cord), and grade 5 neutropenic sepsis]. These adverse events appeared to be related to treatment because all were reversed after treatment discontinuation except for neutropenic sepsis. Neutropenic sepsis, accompanied by nausea and diarrhea, developed in a 51‐year‐old patient (BSA, 1.43 m^2^) who had failed 5 different chemotherapy regimens for metastatic breast cancer prior to study enrollment. Her day 1 AUC for S‐516 was found to be the highest among all the participants. In this patient, grade 4 neutropenia began on day 6 and persisted until day 15 when she died of septic shock. Subsequent dose levels were decreased to dose level 3 (20 mg/d). At this dose level, no additional patients developed DLTs.

**Table 3:** DLT incidence in the dose‐escalation cohort

Cohort	Daily dose (mg)	N	No. patients with DLT
Cohort 1	5	3	0
Cohort 2	10	3^a^	0
Cohort 3	20	6	0
Cohort 4	25	3	3

a One of the four patients in this cohort was removed from the trial during the DLT evaluation period due to early disease progression; thus, only 3 patients were evaluated for a DLT.

### 3.4 Safety profiles in the expansion cohort

Given that CKD‐516 is a VDA, we augmented the expansion cohort with patients who had hypervascular tumors, such as HCC (n = 5) and renal cell carcinomas (n = 3). Patients in the expansion cohort were initially treated with 20 mg CKD‐516/d, which did not cause any DLTs during the dose‐escalation phase. The first patient with HCC (BSA, 1.34 m^2^) treated with this dose, however, developed a DLT (jaundice). As a patient with HCC (BSA, 1.73 m^2^) did not develop DLT after the same dose (20mg) in the dose level 3, the study protocol was then modified so that the daily doses were set at 15 mg/d and 20 mg/d for a BSA less than and greater than or equal to 1.65 m^2^, respectively. This modified BSA‐based dosing regimen was used for the next 10 patients enrolled in the expansion cohort. Of these patients, 7 were evaluated for DLTs, 1 patient withdrew from the trial, and 2 patients were removed from the trial due to early disease progression. Of the 7 evaluated patients, 3 developed a DLT (nausea, thrombocytopenia, and sepsis). Notably, 2 of these 3 patients had HCC accompanied by splenomegaly.

Among the dose‐escalation and expansion cohorts, 11 patients without any evidence of splenomegaly completed at least 1 cycle of a daily CKD‐516 dose of either 15 or 20 mg, depending on BSA status. Of these 11 patients, only 1 patient (9.1%) suffered from any DLT.

### 3.5 Efficacy

Twenty‐two patients were evaluated for drug efficacy (3, 4, 6, and 9 patients for dose levels 1, 2, and 3, and the expansion cohort, respectively). No patients demonstrated any objective responses to CKD‐516, according to the RECIST guideline (7 (31.8%) with stable disease and 15 with progressive disease). Median progression‐free survival was 1.33 months (95% CI: 0.76‐2.23). According to PERCIST guideline,^13^ 8 patients (36.4%) demonstrated stable metabolic disease at week 8.

### 3.6 Pharmacokinetics assessment

**Figure 1:**
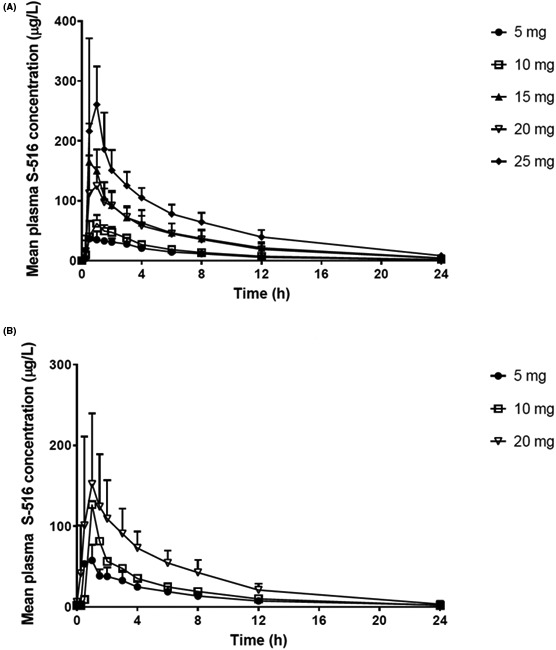
Time‐plasma concentration curves for S‐516. Time‐plasma concentration curves for S‐516 after a single oral administration of CKD‐516 on days 1 (A) and 19 (B)

Day 1 time‐concentration profiles of S‐516 were similar across all CKD‐516 doses (Figure 1A). Median T_max_ levels ranged from 0.5 to 0.75 hours, 0.5 to 1 hours, and 2 to 3 hours for CKD‐516, S‐516, and M9, respectively. Trough levels of S‐516 before day 5 dosing were only quantifiable (33 ng/mL) in 1 of the 23 patients evaluated. Median T_1/2_ levels ranged from 0.44 to 1.42 hours, 4.83 to 5.25 hours, and 7.46 to 16.98 hours for CKD‐516, S‐516, and M9, respectively (Table S1). Ratios of C_max_ and AUC_last_ values of S‐516 between days 1 and 19, which was used to indicate bioaccumulation of metabolites, were 1.26‐1.62 and 1.21‐1.35 for C_max_ and AUC_last_, respectively (Figure 1B).

**Figure 2:**
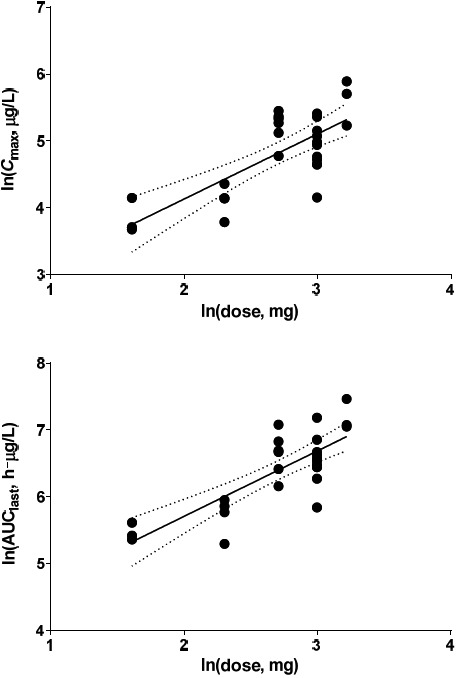
Dose‐C_max_ and dose‐AUC_last_ linear regression profiles of S‐516 after a single oral dose of CKD‐516 on day 1

C_max_ of CKD‐516, S‐516, and M9 had positive correlation to the dose of CKD‐516 [adjusted *R* ^2^ =  .283, .555, and .459; *P* = .0025, <.0001, and <.0001, respectively]. AUC_last_ of CKD‐516, S‐516, and M9 also showed positive correlation to the dose of CKD‐516 [adjusted *R* ^2^ = .349, .623, and .305; *P* = .0007, <.0001, and .0017, respectively]. The 95% CIs of the slope of the log‐transformed parameters included 1 (Figure 2). For CKD‐516, the slope of log‐transformed AUC_last_ was 0.73 (95% CI, 0.34‐1.12). The slopes of log‐transformed AUC_last_ regression lines were 0.98 (95% CI, 0.68‐1.28) and 1.08 (95% CI, 0.45‐1.72) for S‐516 and M9, respectively. Regression analyses of the log‐transformed C_max_ values yielded similar results (95% CIs: CKD‐504, 0.30‐1.24; S‐516, 0.63‐1.32; M9, 0.58‐1.45, respectively). Thus, the exposure to S‐516 (and CKD‐516 and M9) increased with CKD‐516 dose increment in the range 5‐25 mg.

**Figure 3:**
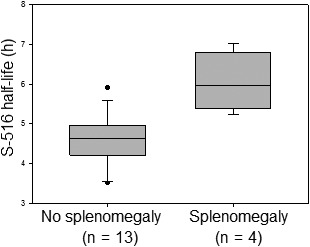
T_1/2_ of S‐516 in patients receiving 15‐20 mg CKD‐516/d with or without radiographic evidence of splenomegaly (*P* = .0016, *t* test). All patients who received at least 1 CKD‐516 dose were analyzed, including those who could not complete the first cycle of CKD‐516 administration due to disease progression. Box plots display 5%, 25%, median, 75%, and 95% T_1/2_ values, with outliers shown as dots

Notably, the T_1/2_ of S‐516 was significantly prolonged in cases showing radiographic evidence of splenomegaly. As shown in Figure 3, the T_1/2_ values of S‐516 in patients receiving 15‐20 mg CKD‐516/d were significantly longer in patients with splenomegaly than those without [6.1 hours vs 4.6 hours, respectively (*P* = .0016, *t* test)] according to the *post hoc* analyses. S‐516 AUC_last_ values also trended higher in patients with splenomegaly than in those without (901 μg*h/L vs 705 μg*h/L, respectively; *P* = .165, *t* test).

## 4 DISCUSSION

An oral formulation of CKD‐516 was developed to improve the drug's anticancer activity and patient compliance. In this study, oral CKD‐516 exhibited manageable toxicity profiles up to the recommended phase II dose (15 mg for patients with a BSA < 1.65 m^2^; 20 mg for patients with a BSA ≥ 1.65 m^2^) in patients without splenomegaly. General toxicity profiles of oral CKD‐516 appeared similar to those of intravenous CKD‐516.^11^ Diarrhea was the most common (13%) grade 3 toxicity associated with weekly intravenous CKD‐516 administration,^11^ consistent with results of this study. Grade 2 or 3 nausea was also very common (30%), but was generally manageable with subsequent serotonin antagonists and steroids. In contrast, oral CKD‐516 was not associated with hypertension, which was the only DLT observed in the intravenous CKD‐516 trial.^11^ Notably, oral CKD‐516 demonstrated no significant cardiovascular adverse events that are observed with other VDAs.^4^, ^5^, ^6^, ^11^, ^14^

One pharmacological benefit of CKD‐516 is its rapid absorption. We observed that the T_max_ of S‐516 was relatively short (0.5‐1 hours), which indicates that oral CKD‐516 is rapidly absorbed and quickly converted into an active moiety. Additionally, C_max_/AUC_last_ profiles of S‐516 after multiple administration of CKD‐516 did not indicate any significant bioaccumulation of active metabolite, S‐516. Exposure of S‐516 at MTD was similar between single oral and intravenous administration of CKD‐516.^11^ Based on a phase I trial of weekly intravenous CKD‐516, the recommended phase II dose of 9 mg/m^2^ (administered on days 1 and 8 every 3 weeks) achieved a mean S‐516 AUC_last_ of 727 μg*h/L.^11^ In this trial, an oral dose of 20 mg/d achieved a mean S‐516 AUC_last_ of 726 μg*h/L, which is approximately 7.5‐fold higher than that of the recommended phase II dose of intravenous CKD‐516 (9 mg/m^2^ on days 1 and 8 every 3 weeks) within a given cycle.^11^ Intravenous CKD‐516 was also tested as a biweekly regimen (NCT01560325) using a dose of 9 mg/m^2^ (days 1, 4, 8, and 11 every 3 weeks; unpublished data). The resulting mean S‐516 AUC_last_ on day 11 of the first cycle was 936 μg*h/L, and the AUC values were comparable between days 1 and 11, without significant S‐516 bioaccumulation (unpublished data). The mean S‐516 AUC_last_ of 726 μg*h/L, which was achieved after an oral dose of 20 mg CKD‐516/d in the current trial, is still approximately threefold higher than that achieved using the recommended phase II dose of biweekly intravenous CKD‐516 within a given cycle. Notably, our serum pharmacokinetics data on day 19 indicated no significant S‐516 bioaccumulation after repeated oral administration.

The S‐516 AUC_last_ of dose level 4, which was associated with high incidence of DLTs, ranged from 1153 to 1743 μg*h/L. A BSA‐based dosing regimen was then designed to target lower AUC_last_ levels based on pharmacokinetics modeling. Pharmacokinetics modeling predicted that the BSA‐based dosing (15 mg (BSA < 1.65 m^2^) or 20 mg (BSA ≥ 1.65 m^2^)) would achieve the S‐516 AUC_last_ ranging from 606 to 802 μg*h/L, which was not expected to cause DLT (Table S2). This value was close to 716‐786 μg*h/L, the range of S‐516 AUC_last_ of subjects who demonstrated stable disease without DLT. Hence, the study protocol was modified so that the daily doses for expansion cohort were 15 mg/d and 20 mg/d for a BSA less than and greater than or equal to 1.65 m^2^, respectively. In the expansion cohort, a single daily dose of 15 mg (BSA < 1.65 m^2^) or 20 mg (BSA ≥ 1.65 m^2^) of CKD‐516 five days per week was tolerable in patients without liver cirrhosis‐associated splenomegaly. HCC‐associated splenomegaly was associated with a longer T_1/2_ of S‐516 and more frequent DLTs. In our expansion cohort, 2 of 3 patients with a DLT showed radiographic evidence of splenomegaly, significantly longer S‐516 T_1/2_ values, and higher S‐516 AUC_last_ values. As S‐516 is metabolized by oxidation, demethylation, and acetylation in the liver, prior to excretion in the bile,^7^, ^8^, ^9^, ^10^, ^11^ reduced hepatic cell mass due to cirrhosis may underlie the accumulation of active S‐516 due to reductions in drug‐metabolizing enzymes. Hence, oral CKD‐516 should be used with extreme caution in patients with liver cirrhosis complicated by portal hypertension. At the time of this writing, oral CKD‐516 is being tested in combination with irinotecan in a phase 1/2a trial in patients with metastatic colorectal cancer (NCT03076957).

## DISCLOSURES

Drs. Soo Jin Kim, Semi Kim, and Keun Ho Ryu are current employees of Chong Kun Dang.

## AUTHOR CONTRIBUTION

HKK, YP, JYK, MK, SJK, SK, KHR, and JP conceptualized the study. JWK, SY, YK, JC, SK, and HKK conducted pharmacokinetic analyses. HKK, KSL, TY, KK, MHK, TK, JC, SJK, KHR, and JP conducted clinical trials and analyzed clinical data. HKK and JP drafted the manuscript.

## Data Availability

Research data are not shared.
