# Supplementary material for: Phase I and pharmacokinetic study of the vascular‐disrupting agent CKD‐516 (NOV120401) in patients with refractory solid tumors
Source: Pharmacol Res Perspect. 2020 Mar 12;8(2):e00568. doi: 10.1002/prp2.568 (PMC7066534; doi:10.1002/prp2.568)
Supplement: Supplementary file 1 [file PRP2-8-e00568-s001.pdf]

Supplementary Tables

Kim H, et al. Phase I and pharmacokinetic study of the vascular-disrupting agent CKD-516 (NOV120401) in patients with refractory solid tumors

Supplementary Table 1. Day 1 S-516 pharmacokinetics.

Dose Group		S-516					
		T_{max} (hr)	C_{max} (ug/L)	AUC_{last} (hr*ug/L)	AUC_{inf} (hr*ug/L)	$t_{1/2}$ (hr)	CL/F (L/hr)
5 mg (n=3)	Mean	1.50	47.90	237.35	245.89	4.83	20.60
	SD	1.32	13.38	32.40	34.98	0.74	2.80
	Min	0.50	39.52	212.72	216.58	4.09	17.57
	Median	1.00	40.85	225.28	236.48	4.85	21.14
	Max	3.00	63.34	274.05	284.62	5.56	23.09
10 mg (n=4)	Mean	1.00	62.10	313.16	326.77	4.64	32.38
	SD	0.00	14.00	80.49	80.11	1.04	9.74
	Min	1.00	44.12	198.97	214.45	3.09	25.00
	Median	1.00	62.98	334.86	346.31	5.07	28.94
	Max	1.00	78.31	383.95	400.03	5.33	46.63
15 mg (n=6)	Mean	0.58	189.02	797.19	833.77	5.04	19.80
	SD	0.20	40.74	248.36	284.67	1.05	6.74
	Min	0.50	118.55	471.87	489.40	4.16	11.46
	Median	0.50	200.76	796.09	815.07	4.77	18.41
	Max	1.00	232.54	1187.21	1309.29	7.01	30.65
20 mg (n=11)	Mean	0.96	141.60	726.39	756.48	4.90	29.53
	SD	0.42	48.39	247.83	267.87	0.85	11.09
	Min	0.50	63.73	343.46	346.00	3.51	14.26
	Median	1.00	140.03	690.06	712.04	4.96	28.09
	Max	2.00	223.63	1316.91	1402.74	6.13	57.80
25 mg (n=3)	Mean	0.83	282.91	1358.83	1424.39	5.25	18.26
	SD	0.29	88.57	333.04	367.69	0.82	4.10
	Min	0.50	186.96	1152.83	1205.42	4.33	13.52
	Median	1.00	300.24	1180.60	1218.86	5.53	20.51
	Max	1.00	361.54	1743.06	1848.89	5.89	20.74

Supplementary Table 2. Pharmacokinetic model-based prediction of S-516 AUC_{last} according to BSA

BSA (m^2)*	Dose (mg/day)	Dose/BSA ($mg/m^2/day$)	Predicted AUC_{last}** (μg*h/L)
1.43	15	10.5	694.23
1.51	15	9.9	657.17
1.57	15	9.6	632.02
1.64	15	9.1	605.55
1.65	20	12.1	802.10
1.70	20	11.8	778.28
1.80	20	11.1	735.26
1.88	20	10.6	704.16

*Several body surface areas are shown as an example based on BSA (1.43–1.88) of 1–4 cohort subjects.

**The AUC_{last} was calculated using 66.2 ± 19.7 μg*h/L /mg/m^2, this value is Dose/BSA-normalized mean AUC_{last} in dose escalation cohorts.
